# Isolation and characterization of two phosphate-solubilizing fungi from rhizosphere soil of moso bamboo and their functional capacities when exposed to different phosphorus sources and pH environments

**DOI:** 10.1371/journal.pone.0199625

**Published:** 2018-07-11

**Authors:** Yang Zhang, Fu-Sheng Chen, Xiao-Qin Wu, Feng-Gang Luan, Lin-Ping Zhang, Xiang-Min Fang, Song-Ze Wan, Xiao-Fei Hu, Jian-Ren Ye

**Affiliations:** 1 Co-Innovation Center for Sustainable Forestry in Southern China, College of Forest, Nanjing Forestry University, Nanjing, Jiangsu, China; 2 2011 Collaborative Innovation Center of Jiangxi Typical Trees Cultivation and Utilization, College of Forestry, Jiangxi Agricultural University, Nanchang, Jiangxi, China; 3 Management School of Nanchang University, Nanchang, China; USDA Forest Service, UNITED STATES

## Abstract

Phosphate-solubilizing fungi (PSF) generally enhance available phosphorus (P) released from soil, which contributes to plants’ P requirement, especially in P-limiting regions. In this study, two PSF, TalA-JX04 and AspN-JX16, were isolated from the rhizosphere soil of moso bamboo (*Phyllostachys edulis*) widely distributed in P-deficient areas in China and identified as *Talaromyces aurantiacus* and *Aspergillus neoniger*, respectively. The two PSF were cultured in potato dextrose liquid medium with six types of initial pH values ranging from 6.5 to 1.5 to assess acid resistance. Both PSF were incubated in Pikovskaya’s liquid media with different pH values containing five recalcitrant P sources, including Ca_3_(PO_4_)_2_, FePO_4_, CaHPO_4_, AlPO_4,_ and C_6_H_6_Ca_6_O_24_P_6_, to estimate their P-solubilizing capacity. No significant differences were found in the biomass of both fungi grown in media with different initial pH, indicating that these fungi could grow well under acid stress. The P-solubilizing capacity of TalA-JX04 was highest in medium containing CaHPO_4_, followed by Ca_3_(PO_4_)_2_, FePO_4_, C_6_H_6_Ca_6_O_24_P_6_, and AlPO_4_ in six types of initial pH treatments, while the recalcitrant P-solubilizing capacity of AspN-JX16 varied with initial pH. Meanwhile, the P-solubilizing capacity of AspN-JX16 was much higher than TalA-JX04. The pH of fermentation broth was negatively correlated with P-solubilizing capacity (*p*<0.01), suggesting that the fungi promote the dissolution of P sources by secreting organic acids. Our results showed that TalA-JX04 and AspN-JX16 could survive in acidic environments and both fungi had a considerable ability to release soluble P by decomposing recalcitrant P-bearing compounds. The two fungi had potential for application as environment-friendly biofertilizers in subtropical bamboo ecosystem.

## Introduction

Moso bamboo (*Phyllostachys edulis* (Carrière) J. Houz.) covers an area of 3.87 million ha, accounting for 70% of the bamboo forest area in China and 80% of its global distribution [1, 2]. It has become the main economic forest crop for satisfying the industrial requirement as well as improving forestry efficiency and farmers’ income [3, 4]. However, due to long-term intensive management, soil fertility has declined and nutrient shortages have restricted the improvement of moso bamboo productivity.

Phosphorus (P) is one of the most important macro elements for plant growth and development. Recent studies have shown that P is the main factor limiting moso bamboo productivity [5–7]. Soil P is classified into inorganic and organic P, and 60–80% of the total P is inorganic P, since P is an element of the sedimentary cycle. According to the P fractionation method provided by Chang and Jackson (1957)[8], the inorganic P was divided into five main groups including soluble P, calcium phosphate, aluminum phosphate, iron phosphate and occluded P. Generally, the soluble P, which can be directly absorbed by plants, accounts for a little proportion in soil, while the rest of inorganic P, such as calcium phosphate, aluminum phosphate, iron phosphate exist with hard-to-dissolve forms [9]. In China, approximately 95% of the P in soil is recalcitrant P, which has been overcome traditionally by adding P fertilizers; however, application of chemical fertilizer has resulted in a number of environmental problems, such as soil pollution and water eutrophication [10]. According to the latest estimates, the global reserves of P could become depleted within 50–100 years [11]. Besides the efficient use of P reserves, it is also important to reduce the current wastage of P fertilizers and to recover applied P.

Phosphate-solubilizing microorganisms are recognized as a solution to the challenges in P fertilization management due to their abilities to mobilize P from recalcitrant sources [12]. In general, phosphate-solubilizing fungi (PSF) possess greater abilities to release P from recalcitrant inorganic P, when compared to bacteria [13]. To date, most representative strains of *Aspergillus* spp. and *Penicillium* spp. have been widely reported as P solubilizers [12, 14], and the stability of P-solubilizing capability of these fungi has been noted to be superior to that of P-solubilizing bacteria upon repeated sub-culturing in synthetic medium [15]. Moreover, PSF have been observed to secrete more acids than bacteria and display greater P-solubilizing activity [13]. Although inoculation of PSF into soil has been reported to increase the level of available P in the soil by mobilizing P fixed in soil particles which results in improved plant growth [16], the abilities of these fungi to function in different environments have not been studied completely, especially in moso bamboo forest conditions with high soil acidity and deficits in available-P [6].

The aim of this study was to isolate and culture fungi that have potential as environment-friendly biofertilizers with the capacity to solubilize recalcitrant phosphates in moso bamboo production areas in China, including, 1) isolation and identification of PSF from rhizosphere soils of moso bamboo; 2) assessment of PSF’s capacity to function in soil acidic environments, from extremely low to neutral pH conditions across moso bamboo production regions, and 3) comparison of the differences in PSF capacity to solubilize various phosphate compounds widely found in the study regions. The results may provide insights into potential microbial mechanisms to explain the wide distribution of moso bamboo in acidic soil and P-deficit areas.

## Materials and methods

### Isolation of PSF

Fifteen soil samples were collected from three moso bamboo production areas (Dagang, Guanshan, and Dajing) in the red soil region of Jiangxi Province, China (S1 Fig), between July and September 2015. The general characteristics of the study samples are shown in Table 1. Five replicate samples were obtained from each site. Each sample comprised the soil adhering to the roots of moso bamboo. The samples were carefully shaken in plastic bags to separate the soil from the roots, and immediately transferred to a cooler until arrival at the laboratory. All samples were stored at 4°C and analyzed within 2 weeks. The soil pH (soil: water = 1:2.5) was determined with a PHS-3C pH meter (Shanghai Lida Instrument Factory, Shanghai, China). Total nitrogen (N) was determined by the Kjeldahl method, and total P was determined by the molybdenum-stibium colorimetry method, respectively, after the samples were digested with sulfuric acid [17]. The research site did not involve endangered or protected species, and no specific permissions were required for conducting experiment.

**Table 1:** Basic parameters and soil characteristics of the three sampling sites.

Sampling sites	Longitude	Latitude	Elevation (m)	pH	Total N (g·kg^-1^)	Total P (g·kg^-1^)
Dagang	114°56'31"E	28°37'17"N	381.0	5.16±0.09a	4.65±0.10a	0.27±0.03b
Guanshan	114°34'47"E	28°33'35"N	548.0	4.37±0.07b	2.51±0.09c	0.33±0.04b
Dajing	114°8'19"E	26°34'8"N	1105.0	4.39±0.06b	3.44±0.08b	0.54±0.07a

Notes: Value = Mean ± standard error. n = 5. The different letters indicated the significant differences among the three sampling sites at the level of *p*<0.05.

Approximately 10 g of each soil sample was transferred to an Erlenmeyer flask containing 90 mL of sterile water and shaken at 120 rpm for 60 min. Subsequently, a series of 10-fold dilutions of the suspension were prepared for each sample, and 200 μL of each dilution was plated on Pikovskaya’ s agar, made from 10 g glucose, 0.2 g NaCl, 0.2 g KCl, 0.5 g (NH_4_)_2_SO_4_, 0.1 g MgSO_4_·7H_2_O, 0.5 g FeSO_4_·7H_2_O, 0.5 g MnSO_4_·7H_2_O, 0.5 g yeast extract, and 18 g agar in 1000 mL of distilled water, and 0.5% tricalcium phosphate as recalcitrant P source [18]. The PSF were identified by the presence of a clear halo around colonies after 8 d incubation at 25°C. The experiments were performed in triplicate. PSF in the samples were isolated, based on the size of halo zones, and purified by repeated culturing on potato dextrose agar (PDA) at 25°C.

### Identification of PSF

PSF characteristics were assessed using Czapek yeast extract agar (CYA) medium containing 30.0 g sucrose, 1.0 g K_2_HPO_4_, 3.0 g NaNO_3_, 0.5 g MgSO_4_·7H_2_O, 0.5 g KCl, 0.01 g FeSO_4_·7H_2_O, 5.0 g yeast extract, 15.0 g agar, and 1 mL of trace elements solution in 1000 mL of distilled water. The trace elements solution contained 1% w/v ZnSO_4_·7H_2_O and 0.5% w/v CuSO_4_·5H_2_O in distilled water. The CYA medium was autoclaved at 121°C for 30 min [19]. Wet mounts were prepared from colonies grown on CYA at 25°C for 7 d and mounted in lactophenol without dye. Microscopic examination and photomicrography were performed with an OLYMPUS BX53 microscope equipped with a MShot image Analysis System (Olympus Corporation, Tokyo, Japan).

DNA extraction was performed following the method of Scott et al. [20]. The ITS region of the nuc rRNA gene was amplified with primers ITS5: 5′-GGAAGTAAAAGTCGTAACAAGG-3′ and ITS4: 5′-TCCTCCGCTTATTGATATGC-3′ [21]; β-tubulin gene (*BenA*) sequences were amplified with primers bt2a: 5′-GGTAACCAAATCGGTGCTGCT TTC-3′ and bt2b: 5′-ACCCTCAGTGTAGTGACCCTTGGC-3′ [22]; and calmodulin gene (*CaM*) was amplified with primers cmdAD2: 5′-GCCGATTCTTTGACCGAGGAAC-3′ and cmdQ1: 5ʹ-GCATCATGAGCTGGACGAACTC-3ʹ [23].

Polymerase chain reactions (PCR) were conducted using 20-μL reaction mixture containing 0.5 μL of each primer (10 pmol/μL), 1.0 μL of genomic DNA (10 ng/μL), 8 μL of 2 × PCR MasterMix buffer (0.05 μg/μL Taq polymerase, 4 mM MgCl_2_, and 0.4 mM dNTPs), and 10 μL of ultrapure sterile water. PCR cycling conditions were as follows: one initial cycle of denaturation at 94°C for 3 min, followed by 34 cycles of 94°C for 30 s, 50°C for 30 s, and extension at 72°C for 45 s, and a final extension for 5 min at 72°C.

After amplification, the PCR fragments were electrophoresed in 2.0% agarose gel with a 100-bp DNA ladder (MBI Fermentas) at 80 V for 20 min, stained in 0.5 μg/mL ethidium bromide water solution for 15 min, and examined by a gel imaging system (BIO-RAD, Gel Doc XR+). Samples with a single obvious band of anticipated length on the gel were purified and sequenced in both directions by Sangon Biotech Co., Ltd., Shanghai, China.

The raw sequences were proofread and edited manually with BioEdit 7.0.9 [24], while the edited sequences were aligned using Clustal W [25] and adjusted manually as required (Table 2). Finally, a neighbor-joining (NJ) phylogenetic tree was constructed with Kimura 2-parameter model to calculate sequence divergence and subjected to 1000 bootstrap replications using MEGA 6.0 [26], with gaps treated as complete deletions.

**Table 2:** Strains with their GenBank accession numbers for three genetic markers.

Strains	Genetic markers		
	ITS	*BenA*	*CaM*
TalA-JX04	MF440335	MF163992	MF375217
AspN-JX16	MF440336	MF163993	MF375216

### Fungal biomass under different pH conditions

Strains TalA-JX04 and AspN-JX16 were grown on PDA plates at 25°C for 6 d, respectively to induce sporulation. Subsequently, the plates were drenched with sterile distilled water, and the spores were carefully removed from the culture surface with an artist’s fine brush. The suspension was then filtered through three layers of sterile cheesecloth to eliminate mycelial fragments. Conidial concentration was determined by a hemocytometer, and adjusted to 10^7^ mL^-1^ by diluting with 0.85% sterile saline. The pH of the potato dextrose liquid medium was precisely adjusted to 1.5, 2.5, 3.5, 4.5, 5.5, and 6.5 with hydrochloric acid, and sterilized at 121°C for 20 min. Then, 0.5 mL each of the spore suspension of TalA-JX04 and AspN-JX16 were inoculated into 30 mL Erlenmeyer flasks with various pH, and incubated at 25°C for 8 d on a shaker. Then, the mycelia were dried at 65°C for 48 h and the dry weights were measured, and the pH values of the fermentation broth after incubation were determined using a PHS-3C pH meter. All experiments were repeated five times.

### P-solubilizing capacity under different pH conditions

After preparation of the conidial suspensions of TalA-JX04 and AspN-JX16, the seed liquid was inoculated into 50-mL flasks containing 30 mL of Pikovskaya’s liquid media (modified by replacing the P sources). Five recalcitrant P sources, including Ca_3_(PO_4_)_2_, FePO_4_, CaHPO_4_, AlPO_4,_ or C_6_H_6_Ca_6_O_24_P_6_, were added to a concentration of 5 g/L, respectively.

The pH of the Pikovskaya’s liquid media was adjusted to 1.5, 2.5, 3.5, 4.5, 5.5, and 6.5 with 2 mol/L hydrochloric acid, respectively, before addition of the P sources, and the medium was sterilized at 121°C for 20 min. Then, 0.5 mL of TalA-JX04 and AspN-JX16 spore suspension was inoculated into 50 mL triangular flasks at various pH and incubated at 25°C and 120 rpm for 8 d on a shaker. The control comprised flask with uninoculated medium. All treatments were centrifuged at 10,000 ×g for 10 min, and the supernatant was used for the measurements of soluble P and pH. The content of soluble P in the supernatant was determined by the colorimetric molybdate blue method [27]. All experiments were performed in triplicate.

### Statistical analysis

All data were subjected to analysis of variance (ANOVA) and the differences among various P sources and pH environments were separated by Tukey test using SPSS package (version 16.0) [28]. Furthermore, a independent-samples t-test was used to identify the difference between TalA-JX04 and AspN-JX16, and the differences were considered significant if *p*≤0.05.

## Results

### Isolation and identification of TalA-JX04 and AspN-JX16

**Fig 1:**
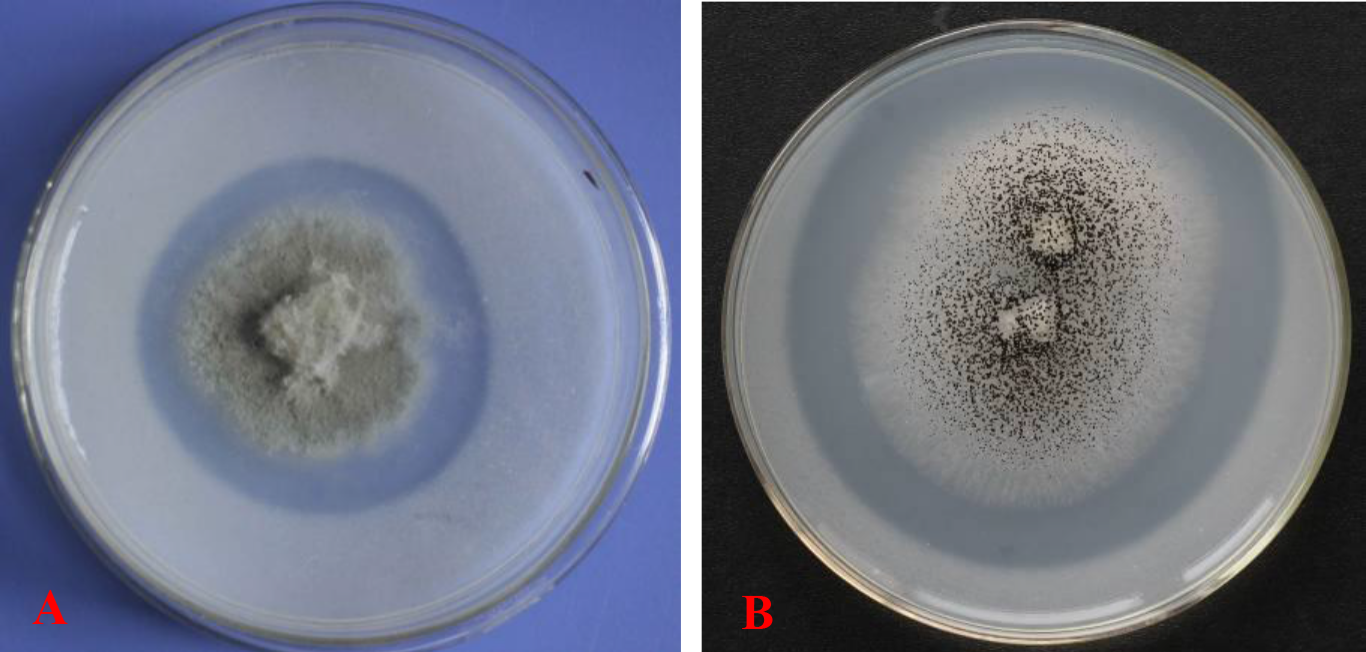
Zones of phosphate solubilization on Pikovskaya’ s agar plates produced by TalA-JX04 (A) and AspN-JX16 (B) incubated at 25°C.

In total, 18 isolates of the moso bamboo rhizosphere fungi were isolated from the three sampling sites, and TalA-JX04 and AspN-JX16 showed a clear zone of dissolved phosphate in solid Pikovskaya’s medium, which indicated that both these PSF exhibited the desired P-solubilizing ability (Fig 1A and 1B).

**Fig 2:**
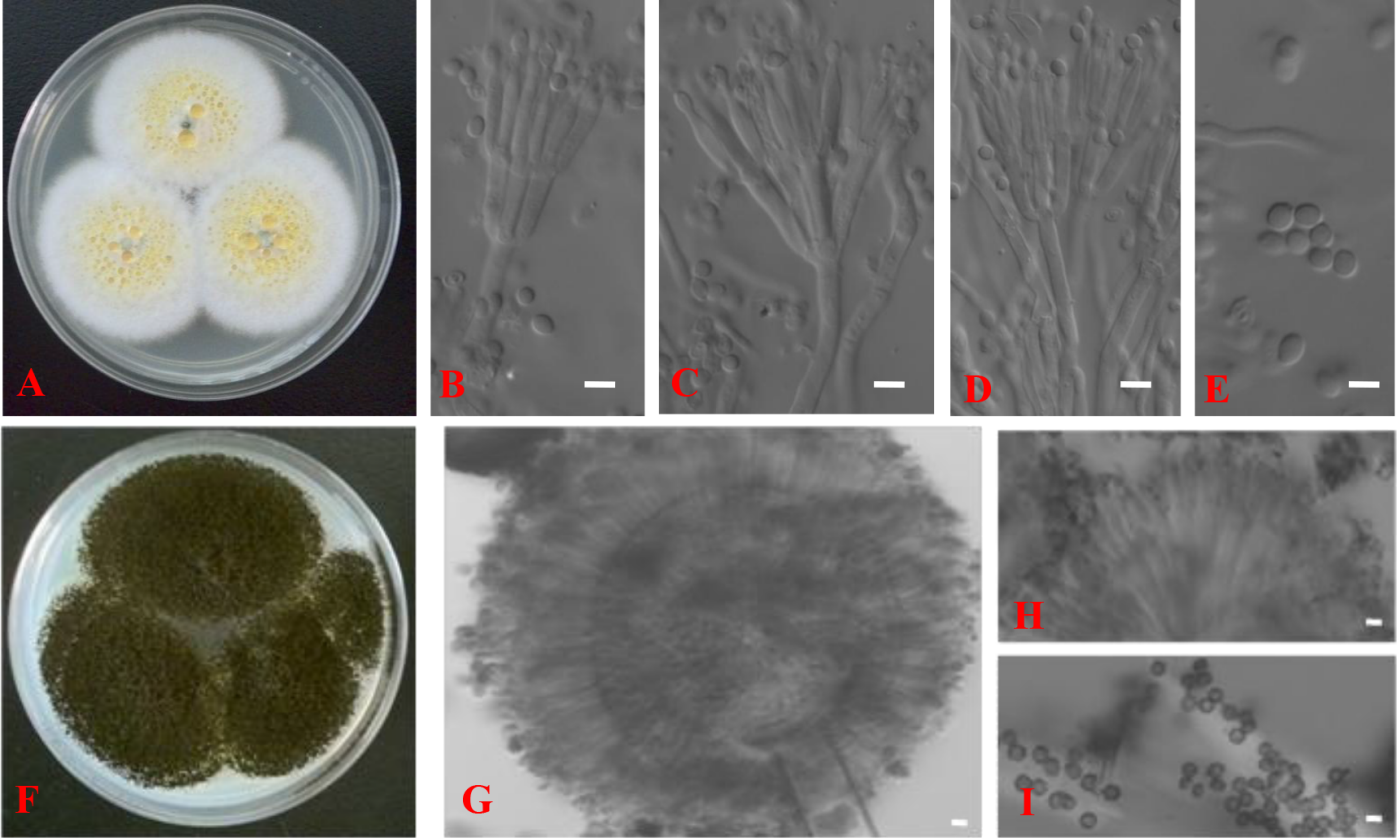
*Talaromyces aurantiacus* (TalA-JX04): Colonies on CYA at 25°C after 7 days (A), Conidiophores, phialides and conidia (B–D); Conidia (E); *Aspergillus neoniger* (AspN-JX16): Colonies on CYA at 25°C after 7 days (F), Head of conidiophore, phialides, and conidia (G); Double spore production cells (H); Conidia (I). Scale bars = 5 μm.

The morphological characteristics of the hyphae, spores, and conidiophores of the two PSF were examined by optical microscopic. TalA-JX04 presented typical penicillate conidiophores with conidia (Fig 2B–2E), whereas AspN-JX16 produced typical double spore production cells, which were identified as the black conidia in conidiophores (Fig 2G–2I).

**Fig 3:**
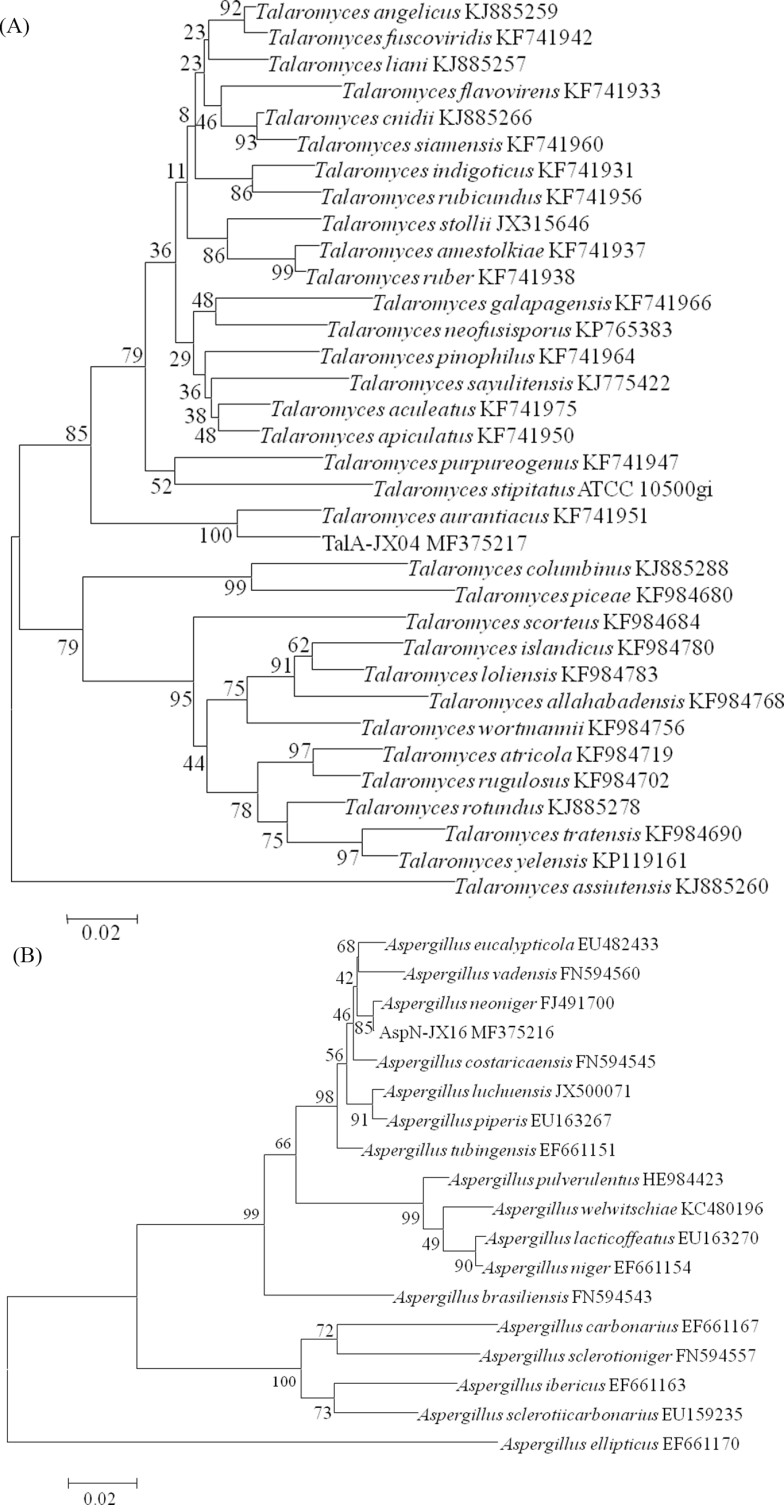
Phylogenetic tree of the *CaM* sequence of TalA-JX04 (A) and AspN-JX16 (B). The NJ phylogram was inferred from partial *CaM* sequence data. Bootstrap percentages of >70% derived from 1000 replicates are indicated at the nodes. Bar = 0.02 substitutions per nucleotide position.

According to the results of ITS, *BenA*, and *CaM* gene sequence analysis to identify the selected PSF, TalA-JX04 was ascertained as *Talaromyces aurantiacus* and AspN-JX16 was confirmed as *Aspergillus neoniger* (Fig 3).

### Mycelial biomass of TalA-JX04 and AspN-JX16 under various pH conditions

Both PSF tolerated to various acidic environments, and the fermentation broth pH showed distinct changes (Table 3). The mycelial biomass of AspN-JX16 was significantly greater at the initial pH of 3.5, when compared to pH 6.5 (*p*<0.05), whereas there was no significant difference in the TalA-JX04 mycelial biomass under various pH conditions (*p*>0.05). Furthermore, no significant difference in the mycelial biomass was observed between TalA-JX04 and AspN-JX16 under each of different pH environments (Table 3). In contrast, the pH of the fermentation broth of both PSF generally increased with increasing initial pH, with TalA-JX04 exhibiting a higher pH increase than AspN-JX16 (Table 3). The pH of the fermentation broth of TalA-JX04 and AspN-JX16 tended to reach 3.44–3.99 and 3.02–3.40, respectively, from the initial pH of 1.5–6.5 (Table 3).

**Table 3:** The mycelial biomass and pH after incubation of TalA-JX04 and AspN-JX16 in the liquid medium under various initial pH.

Initial pH	Mycelial biomass (g L^-1^)		Fermentation broth pH	
	TalA-JX04	AspN-JX16	TalA-JX04	AspN-JX16
1.5	21.42±1.43a	17.46±0.36ab	3.40±0.04c*	3.02±0.09c
2.5	20.31±0.55a	18.33±1.81ab	3.66±0.09b*	3.18±0.06b
3.5	20.55±2.31a	18.77±1.81a	3.71±0.07b*	3.24±0.08b
4.5	22.05±2.32a	17.65±2.25ab	3.71±0.06b*	3.25±0.07b
5.5	22.13±4.42a	16.64±1.42ab	3.88±0.04a*	3.38±0.06a
6.5	22.88±3.71a	16.09±0.87b	3.95±0.11a*	3.40±0.03a

Note: Value = Mean ± standard error. Different letters indicated significant differences (*p*<0.05) among the pH in the same strain. The asterisk (*) indicated the significant difference (*p*<0.05) between TalA-JX04 and AspN-JX16 with the same pH.

### P-solubilizing capacity of TalA-JX04 and AspN-JX16

**Fig 4:**
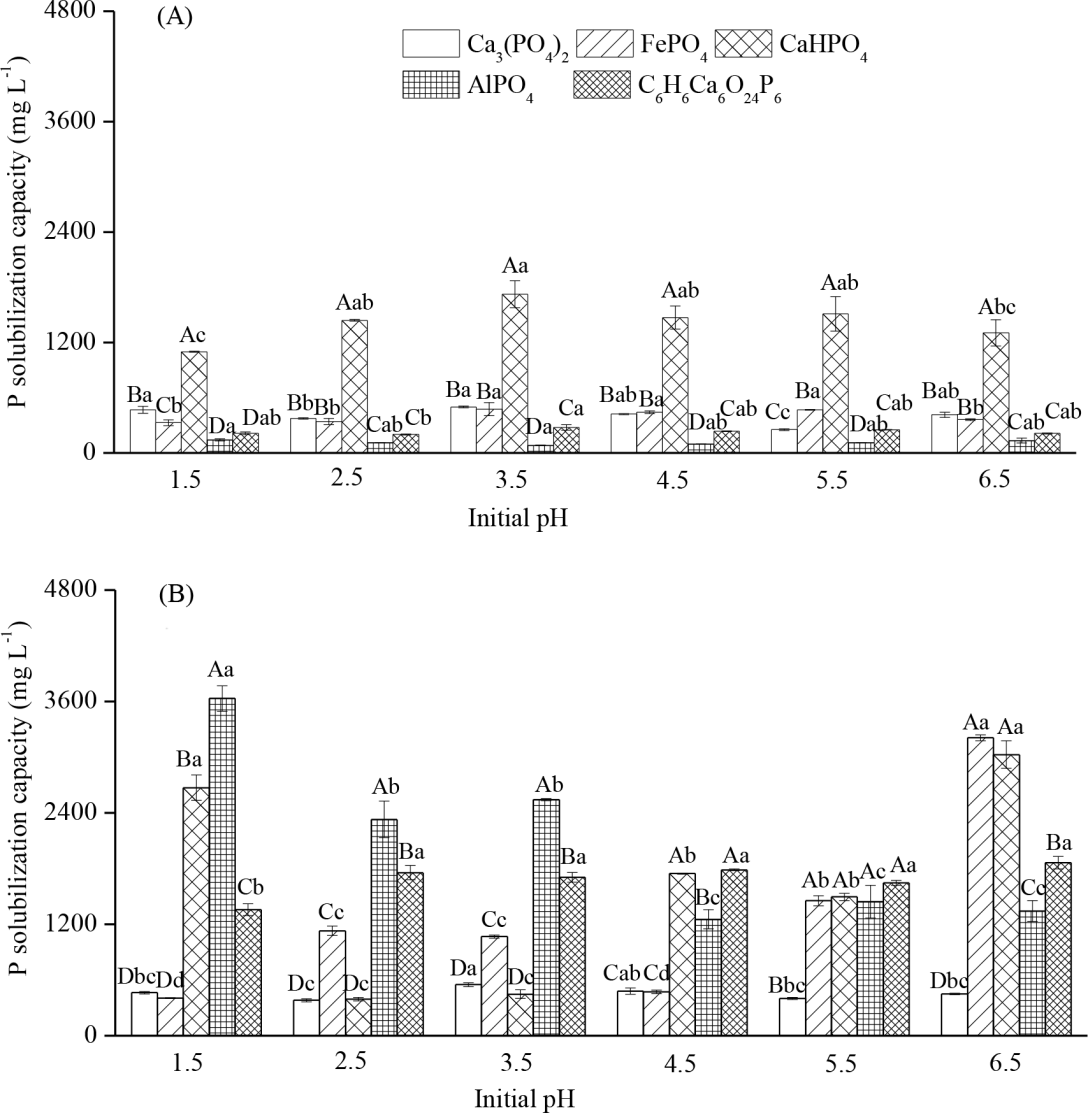
**The solubilizing capacity of five P sources under different initial pH of TalA-JX04 (A) and AspN-JX16 (B).** Note: Value = Mean ± standard error. The uppercase indicated the differences (*p* <0.05) among the five P sources within same initial pH and the lowercase indicated the differences (*p* <0.05) among the initial pH within the same P sources.

The P-solubilizing capacity of TalA-JX04 and AspN-JX16 varied with P source and initial pH environment (Table 4). The P-solubilizing effect of TalA-JX04 was strongest on CaHPO_4_, followed by Ca_3_(PO_4_)_2_, FePO_4_, AlPO_4,_ and C_6_H_6_Ca_6_O_24_P_6_, although interaction between P sources and initial pH environment was observed (Fig 4A). The difference in the P-solubilizing capacity of TalA-JX04 was up to 10 fold among the five different P sources. In contrast, the P-solubilizing effect of AspN-JX16 was influenced by both P sources and initial pH. When exposed to initial pH of 1.5 and 4.5, the P-solubilizing effect of AspN-JX16 was higher on CaHPO_4_, AlPO_4_, and C_6_H_6_Ca_6_O_24_P_6_, when compared to that on Ca_3_(PO_4_)_2_ and FePO_4_. At initial pH of 2.5 and 3.5, the P-solubilizing effect of AspN-JX16 on AlPO_4_ and C_6_H_6_Ca_6_O_24_P_6_ was higher than that on Ca_3_(PO_4_)_2_, FePO_4_, and CaHPO_4_, whereas at initial pH of 5.5 and 6.5, the P-solubilizing effect of AspN-JX16 on Ca_3_(PO_4_)_2_ was lower than that on other P sources (Fig 4B).

**Table 4:** The *F*-values of ANOVA for the effects of initial pH and P sources on phosphate-solubilizing capacity and pH after incubation of TalA-JX04 and AspN-JX16.

Factors	TalA-JX04		AspN-JX16	
	PSC	Liquid pH	PSC	Liquid pH
Initial pH	4.33**	1.88^NS^	91.05***	6.09***
P sources	467.13***	325.58***	370.19***	1090.71***
Initial pH×P sources	2.98**	5.53***	105.33***	4.81***

Note: PSC = Phosphate-solubilizing capacity

^NS^ *p*>0.05

** *p*<0.01

*** *p*<0.001.

The P-solubilizing effects of AspN-JX16 on AlPO_4_ and C_6_H_6_Ca_6_O_24_P_6_ were significantly higher than those of TalA-JX04 at different initial pH environments. Furthermore, at initial pH of ≥3.5, the P-solubilizing effect of AspN-JX16 on FePO_4_ was significantly higher than that of TalA-JX04. However, at initial pH of ≤ 3.5, the P-solubilizing effect of TalA-JX04 on CaHPO_4_ was significantly higher than that of AspN-JX16, whereas the opposite trend was noted at an initial pH of 6.5 (Fig 4).

**Fig 5:**
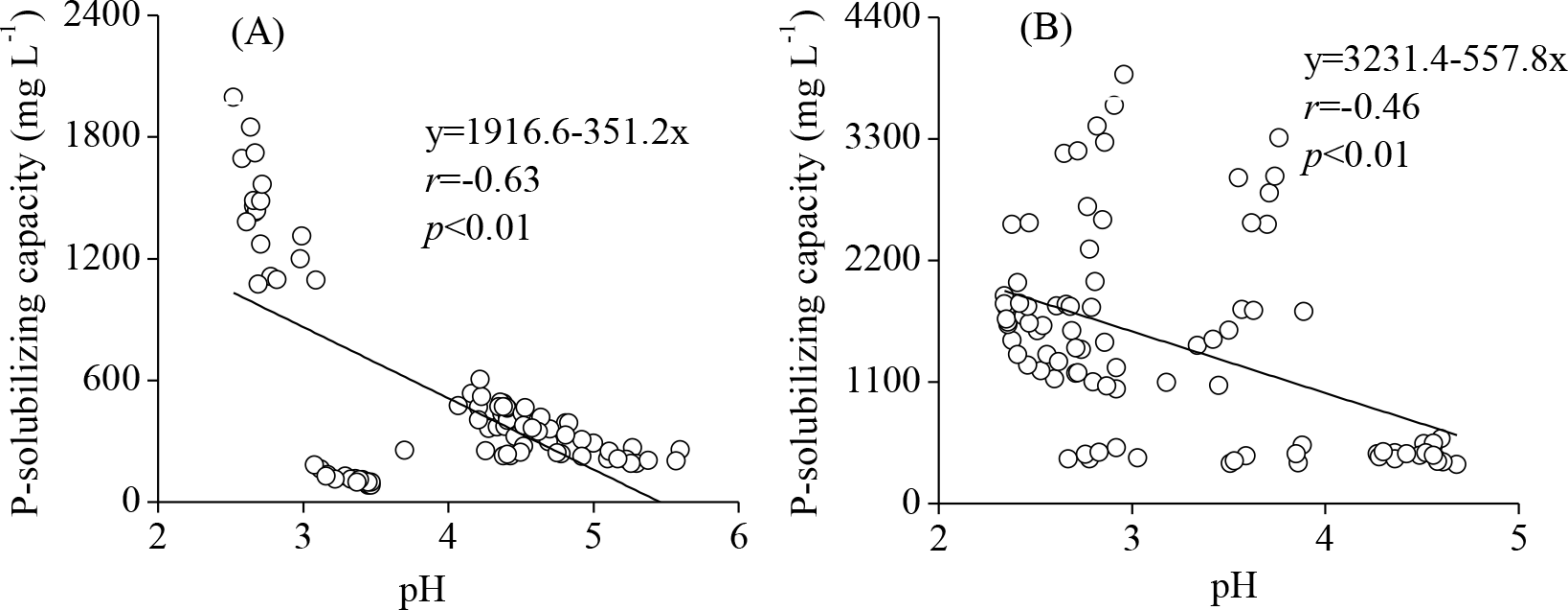
The Pearson’s correlations between the pH of the fermentation broth and P-solubilizing capacity of TalA-JX04 (A) and AspN-JX16 (B).

Meanwhile, the pH of the fermentation broth of TalA-JX04 and AspN-JX16 showed significant changes under different pH conditions, and ranged from an initial pH of 1.5–6.5 to a final pH of 2.5–5.6 and 2.34–4.68, respectively. Correlation analysis demonstrated that the pH of the fermentation broth of the two PSF and solubilized P content were significantly negatively correlated (*r* = −0.63, *p*<0.01 and *r* = −0.46, *p*<0.01 for TalA-JX04 and AspN-JX16, respectively) (Fig 5).

## Discussion

The two PSF, TalA-JX04 and AspN-JX16, were isolated from the rhizosphere soils of moso bamboo in acidic and P-deficit regions. As we know, phosphate solubilization by soil-derived the *Aspergillus* spp., *Penicillium* spp. and their teleomorphs *Talaromyces* spp. had been previously demonstrated [12]. Thus, the two PSF may have the capacity to mobilize and increase moso bamboo’s nutrient uptake. Herein, we demonstrate P-solubiliizing capability using the colorimetric molybdate blue method, the two PSF showed desired P-solubilizing capability in various recalcitrant phosphate. For Ca_3_(PO_4_)_2_, the P-solubilizing ability of TalA-JX04 and AspN-JX16 (about 0.49 and 0.55 g/L, respectively) was much higher than that of P-solubilizing bacteria BK24 (0.37 g/L) isolated from the rhizosphere soil of moso bamboo [29]. The screening of TalA-JX04 and AspN-JX16 could provide useful insights into moso bamboo PSF and may further the development of environment-friendly biofertilizers in P limitation areas.

Our results showed that the mycelial pellet in the culture medium and mycelial biomass of the two PSF did not exhibit significant changes at different initial pH, especially under extreme acidic environments (pH<3.0). Thus the two PSF presented surprising tolerance to extreme pH ranging from 1.5 to 6.5. Although the pH of the fermentation broth of the two PSF generally changed with the increasing initial pH, the fermentation broth pH of TalA-JX04 tended to converge on a narrow pH range (3.02–3.95) from a wide initial pH (1.5–6.5), when compared with that of AspN-JX16. This result showed that organic acids were secreted by the two fungi to adjust the pH of the acidic environment. A previous study reported that the production of organic acids by PSF was different under various initial pH [30]. Thus, both PSF could establish their niches of acidic conditions to maintain their functions, whereas organic acids produced could play a major role in phosphate solubilization [31].

The P-solubilizing ability and the fermentation broth pH of the two PSF differed with various P sources (Fig 4), which demonstrated that the capacity and efficiency of fungi to solubilize P depended on the chemical property of the P source [32]. Previous studies reported that the solubilization of P by *Aspergillus* spp. and *Penicillium* spp. generally decreased the liquid medium pH, which in turn led to the solubilization of P [33]. Thus, the types of P source and the secretion of organic acids by PSF may collectively contribute to the solubilization of P. Organic acids play an important role in phosphate-solubilization processes, which can help the release of P by providing protons and complexing anions, or ligand exchange reactions or complexation of metal ions released to solution. Meanwhile, Scervino et al (2013) found the production of organic acids depended on the interaction of P sources and the fungi [32]. In this study, the pH after incubation was higher in TalA-JX04 than AspN-JX16, while AspN-JX16 showed more efficiency in solubilizing P, compared with TalA-JX04 which may be explained by the finding that the the solubilization of the different P sources mostly depended on the amount of acids production [34].

Although the two PSF were able to solubilize the five types of P sources, the production of soluble P of AspN-JX16 from FePO_4_ and AlPO_4_ was higher compared with that of TalA-JX04. The pH of AspN-JX16 was lower than that of TalA-JX04 after incubation, when they were in Pikovskaya’s liquid medium containing FePO_4_ and AlPO_4_. These results were in agreement with the finding by Barroso et al (2006), who reported that the production of organic acids by *Aspergillus* spp. was higher in the media of AlPO_4_ than other P sources [35]. The PSF secretes various organic acids, such as malic, gluconic, tartaric, oxalic, citric and butyric et al [36, 37]. Li et al found oxalic acid was the main product during FePO_4_ and AlPO_4_ solubilization by *Aspergillus* spp., whereas gluconic acid was mainly produced by *Penicillium* spp. [38]. The lower pH of AspN-JX16 may be attributed to the higher production of oxalic acid, when compared with TalA-JX04.

Moso bamboo is widely distributed over subtropical red soil regions of China [39], and numerous studies demonstrated that the soil pH of moso bamboo stands ranges from 3.7 to 5.2 in southern China [40, 41]. It has been reported that the red soil region of moso bamboo is generally acidic and deficient in most essential nutrients, especially P [5, 42]. As a result, for extensive application of phosphate-solubilizing microorganisms as biofertilizers in these complex soil environments, the microorganisms should be tolerant to a wide range of acidic conditions. Our results showed that TalA-JX04 and AspN-JX16 were tolerant of pH of 1.5–6.5 which was likely beneficial for their growth in acid soils. In addition, both TalA-JX04 and AspN-JX16 displayed high phosphate-solubilizing activity in media containing different recalcitrant P sources. The two isolated PSF have significant potential as biofertilizers in the future.

Usually, red soil is rich in Al^3+^ and Fe^3+^ [43]. Both AlPO_4_ and FePO_4_ have complex structures, when compared with Ca_3_(PO_4_)_2_ [12]. In acidic soils, free oxides and hydroxides of Al and Fe fix P, unlike Ca in alkaline soils [44]. In general, phosphate-solubilizing microorganisms exhibited better P-solubilizing ability in media containing Ca_3_(PO_4_)_2_ and CaHPO_4_, whereas they exhibited low P-solubilizing ability in media containing AlPO_4_ and FePO_4_ [31]. However, the two PSF, especially AspN-JX16, displayed greater P-solubilizing ability in media containing AlPO_4_ and FePO_4_ in addition to other P sources. Therefore, TalA-JX04 and AspN-JX16 are suitable for growth in the acidic environment of moso bamboo stands, however their capacities to release soluble P from red soil needs to be verified since the P sources available in aqueous suspension are generally more easily utilized by fungi than their corresponding molecules bound in soils.

## Conclusions

In this study, we first isolated two phosphate-solubilizing fungi (PSF) from moso bamboo rhizosphere soil in acid soil of Jiangxi province, China. The two PSF, TalA-JX04 and AspN-JX16, were identified as *Talaromyces aurantiacus* and *Aspergillus neoniger* respectively, based on morphologic and phylogenetic characteristics. The mycelial biomass of TalA-JX04 and AspN-JX16 were not inflenced by acidic environment. Meanwhile, the two PSF had considerable abilities to solubilize recalcitrant calcium, aluminum, iron phosphates and phytate in acidic liquid medium. The results showed the secretion of organic acids by PSF should be the key mechanism of phosphate solubilization. Both PSF have the potential for application as environment-friendly biofertilizers in moso bamboo plantations with acidic soils in China.

## Supporting information

S1 Fig The location of the sampling sites in jiangxi province, China. (DOC)
