# Supplementary figures and images for: Isolation and characterization of two phosphate-solubilizing fungi from rhizosphere soil of moso bamboo and their functional capacities when exposed to different phosphorus sources and pH environments
Source: PLoS One. 2018 Jul 11;13(7):e0199625. doi: 10.1371/journal.pone.0199625 (PMC6040707; doi:10.1371/journal.pone.0199625)

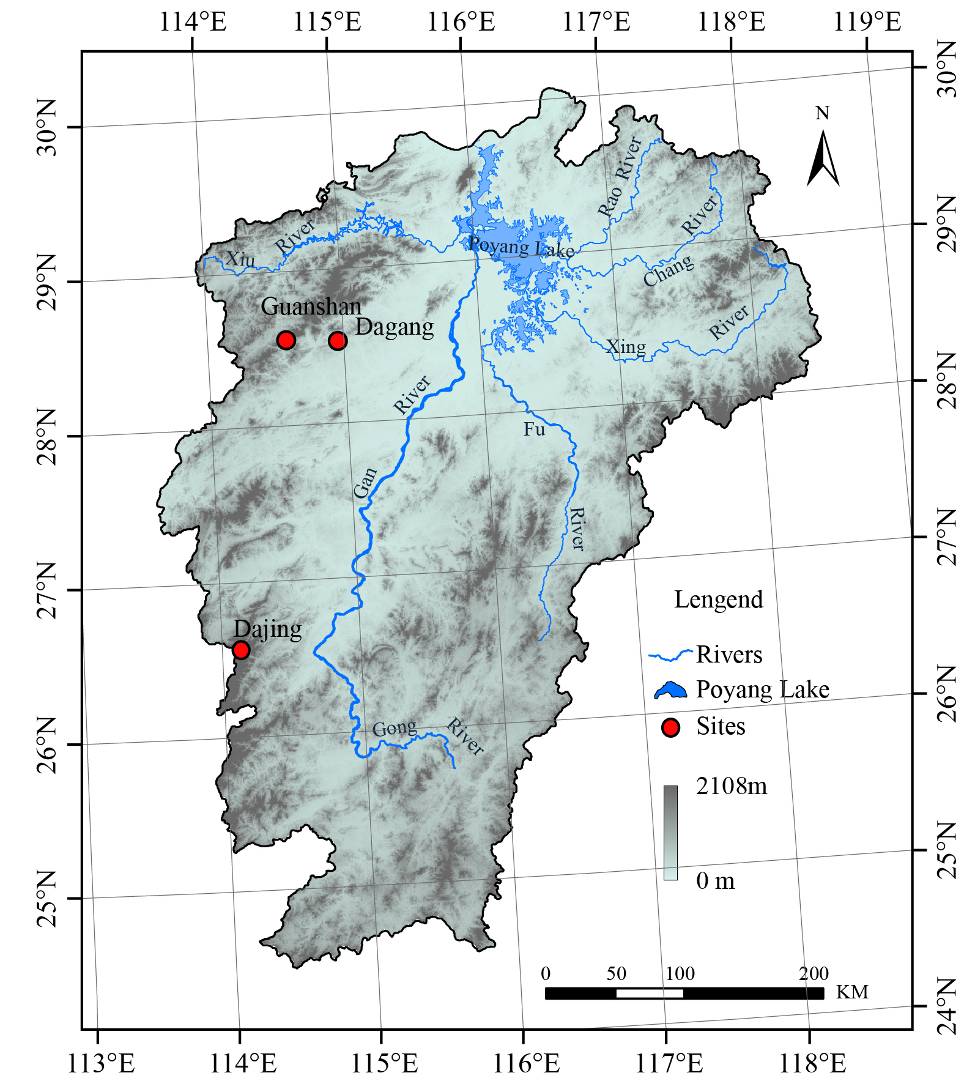


S1 Fig. The location of the sampling sites in Jiangxi Province, China

Supplement: S1 Fig — (DOC) [file pone.0199625.s001.doc]
